# The value of position-specific priors in motif discovery using MEME

**DOI:** 10.1186/1471-2105-11-179

**Published:** 2010-04-09

**Authors:** Timothy L Bailey, Mikael Bodén, Tom Whitington, Philip Machanick

**Affiliations:** 1 Institute for Molecular Bioscience, The University of Queensland, Brisbane 4072, Queensland, Australia

## Abstract

**Background:**

Position-specific priors have been shown to be a flexible and elegant way to extend the power of Gibbs sampler-based motif discovery algorithms. Information of many types–including sequence conservation, nucleosome positioning, and negative examples–can be converted into a prior over the location of motif sites, which then guides the sequence motif discovery algorithm. This approach has been shown to confer many of the benefits of conservation-based and discriminative motif discovery approaches on Gibbs sampler-based motif discovery methods, but has not previously been studied with methods based on expectation maximization (EM).

**Results:**

We extend the popular EM-based MEME algorithm to utilize position-specific priors and demonstrate their effectiveness for discovering transcription factor (TF) motifs in yeast and mouse DNA sequences. Utilizing a discriminative, conservation-based prior dramatically improves MEME's ability to discover motifs in 156 yeast TF ChIP-chip datasets, more than doubling the number of datasets where it finds the correct motif. On these datasets, MEME using the prior has a higher success rate than eight other conservation-based motif discovery approaches. We also show that the same type of prior improves the accuracy of motifs discovered by MEME in mouse TF ChIP-seq data, and that the motifs tend to be of slightly higher quality those found by a Gibbs sampling algorithm using the same prior.

**Conclusions:**

We conclude that using position-specific priors can substantially increase the power of EM-based motif discovery algorithms such as MEME algorithm.

## Background

Short, linear sequence motifs in protein or nucleic acid sequences are of considerable interest to biologists. This type of sequence pattern is often indicative of important biological sequence signals such as transcription factor binding sites (TFBSs) or splice junctions in nucleotide sequences, and of sumoylation sites and stabilization domains in proteins. Consequently, there has been long and continuing interest in developing software algorithms that can automatically discover functional sequence motifs in sets of biopolymer sequences suspected to harbor one or more common sequence signals.

Biological sequence motifs are often quite subtle, and discovering them in a set of sequences is often impossible since real motifs may be indistinguishable from random artifacts. This has encouraged the development of specialized motif discovery algorithms that can effectively utilize information in addition to the sequences themselves.

One successful approach for improving motif discovery using auxiliary data has been to incorporate evolutionary *conservation *information into the discovery process [1,2]. This approach typically augments the set of input sequences with one or more phylogenetic relatives of each of the original sequences. Such motif discovery algorithms are designed to emphasize motifs that are conserved across related species, on the assumption that such motifs are more likely to be functional.

Another fruitful approach has been to utilize biological information to select a "negative" set of sequences, and to modify the search process to seek motifs that are relatively over-represented in the "positive" sequences. This second approach can be also viewed as discovery of *discriminative *motifs [3,4]. Using negative sequences has the effect of steering the motif discovery process away from sequence patterns that are due to effects unrelated to the particular type of motif being sought. This is desirable when searching for binding site in genomic sequences due to the extremely non-random nature of genomic DNA.

A third approach for improving motif discovery has been to seek motifs whose presence in sequences is correlated with some biological signal such as mRNA level. These approaches typically use a *regression *model, and look for motifs that minimize the residual error between a biological signal associated with each input sequence and a motif-based mathematical model of the signal [5,6].

Recently, a general approach has been proposed that allows the incorporation of almost any type of auxiliary information into the class of motif discovery algorithms based on Gibbs sampling [7]. The additional information is converted into a measure of the likelihood that a motif starts at each position in each sequence in the input. This is measure is referred to as a "position-specific prior" (PSP). Gibbs sampling algorithms optimize a Bayesian sequence model, and the PSP approach allows the (summarized) auxiliary information to bias the optimization toward real motifs.

The PSP approach has several advantages. Firstly, it can directly and simultaneously incorporate multiple types of auxiliary data into motif discovery. Secondly, it cleanly separates the problem model optimization from any issues arising from trying to incorporate heterogenous data into the biological sequence model. Thirdly, the PSP methodology can sometimes avoid the severe increase in computational complexity suffered by many of previous approaches to incorporating auxiliary information into motif discovery.

The PSP approach has shown great promise in extending the power of Gibbs sampling-based motif discovery algorithms [7]. For example, a "discriminative conservation" () prior has been shown to be extremely effective for discovering TFBS motifs in yeast sequences when used with a Gibbs sampling algorithm [8]. Incorporation of nucleosome positioning and transcription factor structural class information into a PSP has also proved useful in the discovery of yeast TFBS motifs [9]. However, the benefits of PSPs to EM-based algorithms (such as MEME) has yet to be studied.

In this paper, we describe extending MEME to enable it to use position-specific priors. Like Gibbs sampling-based algorithms, the popular MEME motif discovery algorithm [10] uses a Bayesian probabilistic model in the search for motifs. To allow comparison with previous work, we study the affect of using the  PSP with MEME. This PSP combines evidence of evolutionary conservation with the ability of a motif to discriminate between sequences binding the TF and those that do not.

To explore the benefits of using PSPs with MEME, we focus on discovering TFBS motifs in chromatin immunoprecipitation (ChIP) data for yeast and mouse transcription factors. We show that using the  PSP greatly improves MEME's ability to discover motifs in an extremely well-studied example of 156 sequence sets derived from TF ChIP-chip (ChIP followed by microrarray) experiments in yeast. In fact, using this PSP, MEME discovers the correct TF motif in more of the yeast ChIP-chip datasets than six other algorithms that use conservation information, including the Gibbs sample using the same PSP. We further show that using the  PSP, MEME discovers more *accurate *motifs in mouse ChIP-seq (ChIP followed by sequencing) data [11].

## Methods

We describe the enhancements to MEME required for reading in and utilizing a file containing a position-specific prior corresponding to the input DNA or protein sequences. We cover how PSP information is utilized during each of MEME's three major phases. We also describe how MEME converts a prior on motifs of a width *w*_0 _to a prior on motifs of width *w *≠ *w*_0 _in order to allow MEME to discover motifs of a width different than that for which the prior was derived. (Further implementation details are given in Additional file 1.)

### Incorporating position-specific priors into MEME

The basic task of biological sequence motif discovery is, given a set of DNA or protein sequences, to determine which positions in the sequences are motif occurrences (sites). MEME does this using a statistical sequence model that it creates based on certain hints provided by the user about the number of sites expected in each sequence and the width of the motif sites. The parameters of the model are referred to here collectively as *ϕ*. MEME discovers motifs by optimizing the statistical parameters of the model using the Expectation Maximization (EM) algorithm. The statistical parameters of the model include a position-specific probability matrix (PSPM) representation of the motif, referred to here as *θ*. The PSPM specifies the probability of each possible letter (amino acid or nucleotide) at each of the *w *positions in the motif. A motif is a model of aligned words of a specific length *k *(*k*-mers), each from a different sequence, representing the likelihood of a given letter appearing at each position.

To discover a motif, MEME proceeds in three phases. In Phase I, MEME determines good *starting points *for the EM algorithm. Since MEME automatically determines the width of the motif and the number of sites, this first phase actually selects starting points for various combinations of motif width and number-of-sites. In Phase II, MEME runs the EM optimization algorithm from each starting point determined in the first phase in order to produce a candidate PSPM representation of the motif. In Phase III, MEME scores each of the candidate motif PSPMs found by EM. To do this, it uses the candidate motif PSPM to predict motif sites, and calculates the statistical significance of the relative entropy of the predicted sites. MEME outputs the candidate motif with the highest statistical significance. Once the motif has been selected and output, MEME "probabilistically erases" the motif's sites, and begins the process again at Phase I in order to discover further motifs.

We describe below how we have enhanced MEME to utilize PSP information in each of its three phases. In what follows, we assume that MEME has been provided with a set of *n *sequences **X **= {*X*_1_, ..., *X*_*n*_}. For notational convenience we assume, without loss of generality, that all input sequences have the same length, *L*.

#### Overview of position-specific priors

By default, MEME assumes that every position in every sequence is equally likely *a priori *to be a motif site. Position-specific priors allow the user to change this assumption, thereby causing the sequence model to favor motifs that include high-probability sites over those that do not. A PSP defines, for each position in a given set of *n *sequences, our prior belief that a site (for any motif) starts at that position. To express this in notation, we introduce the binary "missing information" variables **Z **= {*Z*_*i*,*j*_}, where *Z*_*i*,*j *_= 1 if a site starts at position *j *in sequence *X*_*i*_, and *Z*_*i*,*j *_= 0 otherwise. We can then specify a PSP completely by the set of values **P **= {*P*_*i*,*j*_}, where

For convenience, we define the special value *P*_*i*,0 _to be the prior probability there is *no *motif site anywhere in sequence *X*_*i*_. To complete our definition of what a PSP is, we add the assumption that a PSP is tied to a particular motif width, *w*_0_. Therefore, the meaning of *P*_*i*,*j *_is the prior probability of any motif *of width w*_0 _having a site at position *j *in sequence *X*_*i*_. (We discuss later how MEME derives PSPs of different widths from a fixed-width PSP given in its input.)

MEME only allows sites that fit completely within a sequence, so we require that the last *w *- 1 positions in a sequence have *P*_*i*,*j *_= 0. MEME can require every sequence to have one site (OOPS sequence model) or it can allow sequence to have zero or one sites (ZOOPS sequence model). Clearly, this implies (based on our definition of *P*_*i*,0_, above) that *P*_*i*,0 _= 0 in the OOPS sequence model. For the ZOOPS model, we allow *P*_*i*,0 _to have any value in the range [0, ..., 1]. MEME has one more model–the ANR model–that allows any number of motif sites any sequence. We have not yet implemented PSPs for this model.

MEME searches for motifs in the protein or DNA sequences given in its input. However, MEME can also search for DNA motifs that may have sites on either strand. In that case, we index the sites on the opposite strand from -*L *to -1 and we then define *Z*_*i*,*j *_and *P*_*i*,*j *_for *j *∈ [-*L*, ..., *L*]. In order for the *P*_*i*,*j *_to define a probability distribution, they must all lie in the the range [0, ..., 1] and, for the OOPS and ZOOPS sequence models they must sum to 1 for *i *= 1, ..., *n*, where *n *is the number of sequences in the input to MEME. For all sequence models, the sum over site position, *j*, runs from 0 to *L *(rather than -*L *to *L*) in the protein and single-stranded DNA cases. Note that we define *P*_*i*,*j *_= 0 for all values of *j *where a motif would not fit entirely within the sequence.

Our implementation of PSPs in MEME has one additional constraint. When we are considering motifs that may occur on either DNA strand (the strand given in the input sequences or its reverse complement), we require that the PSP be *symmetrical*. That is, we require that *P*_*i*,*j *_= *P*_*i*,-*j *_for all sequences *X*_*i *_and sequence positions *j*. This restriction seems reasonable to us, since the prior probability of any DNA motif in a set sequences by definition is the same as that of its reverse complement motif.

#### Providing position-specific priors to MEME

MEME can now read PSPs in a format described in Additional file 1. When a PSP file is not provided, MEME assumes, as before, a uniform prior over motif site positions. PSPs can be generated using Hartemink software [8] followed by conversion to MEME's PSP format as described in Additional file 1. The MEME PSP format requires that the set of prior values, {*P*_*i*,*j*_} for *i *= 1, ..., *N *and *j *= 0, ..., *L*, be specified, and that they obey all the constraints described above. For any sequences for which priors are not supplied in the PSP file, priors are calculated as uniform priors. The MEME PSP format includes the width, *w*_0_, of the motifs for which the prior is designed. If MEME is run in double-stranded mode on DNA, the symmetry restriction allows us to generate the PSP for the second strand automatically.

#### Renormalizing position-specific priors for motifs of different widths

As mentioned above, a PSP is tied to a particular motif width for which it is derived. When MEME is considering motifs of width *w*, different from *w*_0_, the one specified in the PSP input file, it renormalizes the PSP. The renormalization attempts, in a heuristic fashion, to extend the information captured by the PSP about motifs of width *w*_0 _to a PSP suitable for motifs of width *w*. Renormalization also insures that the new PSP obeys all of the constraints described above. In particular, when *w *>*w*_0_, there are fewer legal positions for motif sites in a given sequence, so the constraint that the *P*_*i*,*j *_sum to 1 would be violated without renormalization.

For motifs that are wider than the width for which the input PSP was designed, the renormalized PSP uses the geometric mean of *P*_*i*,*j *_for all width-*w*_0 _sites that are completely contained by a width-*w *site. The intuition behind this definition is that the information in each of the completely contained sites should be included in our estimate of the prior probability of the longer site containing them. When *w *>*w*_0_, a width-*w *site at position *j *completely contains width-*w*_0 _sites starting at positions *j *through *j *+ *w *- *w*_0_. If we let *c *= *w *- *w*_0 _+ 1 be the number of shorter sites a longer site contains, our renormalized PSP, PSP_(*w*)_, is computed as(1)

for *i *[1, ..., *n*] and *j *∈ [1, ..., *L *- *w *+ 1]. To keep computation costs reasonable, and because the value of information contained in a prior on shorter motifs decreases as the width of the longer motif increases, we constrain *c *≤ *w*_0 _in Eqn. 1.

For motifs that are shorter than those for which the PSP was designed (*w *<*w*_0_), MEME does not renormalize the input PSP. In this case, it simply uses the input PSP as though it were designed for the shorter width motifs, setting PSP_(*w*) _= PSP. This has the implication that some potential motif sites at the ends of sequences will be ignored when searching for shorter motifs, since their *P*_*i*,*j *_values will remain zero even though they are legal starting positions for the shorter motif. For example, if the PSP width is 8 and MEME is searching for motifs of width 7, the last possible position for a motif in each sequence will have *P*_*i*,*j *_= 0. This seems more sensible than setting the value of *P*_*i*,*j *_based, say, on the value of *p*_*i*,*j*-1 _since the width-8 PSP contains no explicit information on the prior probability of a site starting at position *j*. This is because removing the first letter of the word starting at position *j *- 1 might result in a word with a much lower prior probability. In any case, we expect that useful priors will tend to be relatively short (6 to 10) in relationship to the lengths of the sequences containing the motifs. In what follows, we always assume that the PSP has been normalized to the current motif width being considered by MEME, so we drop the width notation from PSP_(*w*) _and *P*_*i*,*j*,*w*_.

#### MEME Phase I: Finding Starting Points

To find starting points for EM, MEME converts each subsequence of the data into a "starting" PSPM and calculates a score for it using an algorithm that approximates one step of EM followed by the scoring phase. Creation of the starting PSPM from a subsequence has been previously described [10]. Each such PSPM, *θ*_*M *_is then used to calculate the probability under the motif model of every potential site in the input sequence, *Pr*(*site*|*θ*_*M*_). Previously, for the OOPS and ZOOPS models, the single site with the highest likelihood from each sequence was determined. For the OOPS model, these sites were then assigned a score. For the ZOOPS model, these sites were sorted in decreasing order by their likelihoods, and the top *t *sites for successively larger values of *t *were scored.

To incorporate PSPs into this phase of MEME, sites are now sorted by a value proportional to their posterior probabilities, *Pr*(*site*|*θ*_*M*_)*Pr*(*θ*_*M*_), where *Pr*(*θ*_*M*_) is the prior probability of the potential site being a real site, as specified by the PSP. That is, if the site starts at position *j *in sequence *X*_*i*_, then *Pr*(*θ*_*M*_) = *P*_*i*,*j*_. We found this approach was not sufficient to insure that the best starting points for EM were found, but that incorporating the PSP into *scoring *the sets of sites with the highest posterior probabilities helped significantly (data not shown). Consequently, the prior probability of each site is now used by MEME when it scores the predicted sites, as described in the next paragraph.

The final score for a potential starting point is a weighted version of the log likelihood ratio (LLR) of its set of predicted sites. The LLR of a set of sites is normally computed by aligning the sites, counting the number of times each letter occurs in each column of the aligned sites, and normalizing the counts to frequencies. To calculate the weighted LLR, MEME scales the individual priors independently in each sequence so that the largest of *P*_*i*,*j *_in each sequence is 1. These scaled priors are then used as weights on the counts of the numbers of letters in each column of the motif.

In more detail, the weighted LLR is computed by MEME as follows. First, MEME computes weights

where  is the maximum value of *P*_*i*,*j *_in sequence *X*_*i*_. The weighted count, *c*_*a*,*k*_, of letter *a *in position *k *of the motif, is computed by adding the weight , 0 ≤  ≤ 1, to *c*_*a*,*k *_when the site at position *j *in sequence *X*_*i *_has letter *a *in position *k *of the site. Thus, sites with higher prior values will contribute more to the weighted counts than sites with low prior values. (Note that, with the uniform prior, all the weights  are 1, so this results in the *c*_*a*,*k *_being unweighted counts.) The weighted counts are then turned into weighted frequencies by dividing by *N*_*wt*_, *f*_*a*,*k *_= *c*_*a*,*k*_/*N*_*wt*_, where *N*_*wt *_is the sum of the weights, , of all sites included in the alignment. We now define a new motif model in terms of parameters  = {*f*_*a*,*k*_}. Let *p*_*a *_= *Pr*(*a*|*θ*_*B*_) be the probability of letter *a *under the zero-order Markov background model supplied as an input to MEME (the default if none is supplied is a zero-order model based on the letter frequencies of the sequence data). If the weights were all equal to 1, the LLR of the set of sites under this new model would be(2)

We refer to Eqn. 2 as the "weighted LLR" of the set of sites when the weights on the sites are not all equal to 1.

For each potential starting PSPM, MEME computes the LLR using Eqn. 2 on different numbers of predicted sites, *t*. MEME does this by considering only the *t *predicted sites with the largest posterior probabilities for successively larger values of *t*. For the OOPS model, the only value of *t *tried is the number of input sequences, *t *= *n*.

MEME repeats this entire process for successively larger values of *w*. For each combination of *t *and *w*, MEME runs EM using the potential PSPM that has the largest weighted log likelihood ratio. EM is described in the next section.

#### MEME Phase II: Expectation Maximization

MEME uses EM to maximize the expectation of the joint likelihood of the sequence model given the sequences **X **and the *missing information *variables **Z **(refer to Table 1). EM proceeds by iterating an E-step followed by an M-step. The only change required to MEME's existing EM implementation is the replacement of uniform assumption of site positions with the position-specific prior in the E-step.

**Table 1:** Definition of terms used in describing the MEME algorithm

*n*	number of input sequences
*L*	length of input sequences

**X **= {*X*_1_, ..., *X*_*n*_}	the set of *n *input sequences

*w*	width of a MEME motif

*m *= *L *- *w *+ 1	number of positions for a site

*γ*	probability of a site in any sequence

*θ*	PSPM model of motif;

**P **= {*P*_*i*,*j*_}	position-specific prior (PSP)

*w*_0_	width for which input PSP is defined

**Z **= {*Z*_*i*,*j*_}	missing information variables for *i *∈ [1, *n*],*j *∈ [-*L, L*]

*Z*^(*t*)^	expectation of **Z **at EM iteration *t*

= *Pr*(*Z*_*i*,*j *_= 1|*ϕ*^(*t*)^)	prior probability given PSP & model

*ϕ*^(*t*)^	model parameters at EM iteration *t*

*ϕ *= {*θ*, *γ*, **P**}	all sequence model parameters

For OOPS and ZOOPS models, the parameters of the sequence model are *ϕ *= {*θ*, *γ*, **P**}. EM re-estimates the PSPM, *θ*, but holds fixed the PSP, **P**. The additional parameter, *γ*, represents the probability that a randomly chosen sequence in the dataset contains a motif site. This is always equal to 1 for the OOPS model, and is estimated by EM for the ZOOPS model.

The E-step of EM computes new estimates of the conditional probabilities of the missing variables **Z**, conditioned on the current estimate of the model parameters,(3)

where *ϕ*^(*t*) ^is the parameter estimate at the start of the current iteration, *t*, of EM. The current estimate of the probability of each possible site based only on the model is *Pr*(*Z*_*i*,*j *_= 1|*ϕ*^(*t*)^). For notational convenience, we define variables that represent this probability for *j *∈ [1, ..., *L*],

where *m *= *L *- *w *+ 1 is the number of places a motif site will fit in a sequence.

With these definitions, the computation in the E-step of the new estimates of the conditional probabilities of missing variables **Z **for the OOPS and ZOOPS models can be written as(4)

for *i *∈ [1, ..., *n*] and *j *∈ [0, ..., *m*]. When searching both DNA strands, the sum in the denominator in Eqn. 4 goes from -*m *to *m*, and we define  for *j *∈ [-*m*, ..., 0, ...,*m*].

The M step re-estimates *ϕ *by solving(5)

The M-step of the EM algorithm in MEME is unchanged. See Bailey and Elkan [10] for more details on how the terms in Eqn. 4 and Eqn. 5 are computed.

#### MEME Phase III: Scoring the Motifs

The scoring phase of the MEME algorithm assigns scores to the motifs discovered by EM. The criterion is based on the statistical significance of the log-likelihood ratio (Eqn. 2) of the most likely sites for the motif in the sequence dataset. Unlike the starting point phase (Phase I), the scoring phase of MEME computes the *unweighted *LLR, even when using non-uniform positional priors. This choice was motivated by tests which showed that the *weighted *LLR performed no better, so we chose to keep this part of the MEME algorithm unchanged (data not shown). Although the scoring phase of MEME was not changed as a result of incorporating PSPs, it has not been documented previously, so we describe it briefly here.

The significance measure used to rank motifs takes into account the LLR of the motif, its width and the number of sites it contains. The sites of a candidate motif are those with the largest final values of **Z**^(*t*)^. For the OOPS model, MEME scores the motif consisting of these sites. With the ZOOPS model, MEME sorts the sites by decreasing *Z*_*i*,*j *_value, and scores each prefix of the sorted list.

MEME scores a motif consisting of a set of sites as follows. The LLR of each column of the aligned sites is computed, and the *p*-value of the column-LLR is computed based on the background Markov model using the dynamic programming method of Hertz and Stormo [12]. These *p*-values are then multiplied together and the *p*-value of the resulting product is computed as described in Bailey and Gribskov [13]. (Computing this column-LLR based *p*-value requires far less time than directly computing the *p*-value of the total LLR of the motif.) To make the scores of various motif widths and numbers of sites compatible, MEME multiplies the *p*-value of the motif by the number of possible ways to select positions for the given number of sites in the set of sequences, **X**. This final score is referred to as the *E*-value of the motif.

### Measuring the Benefits of using PSPs

To evaluate the benefit of using PSPs in motif discovery, we search for motifs in sets of sequences predicted to bind different TFs in yeast and in mouse. The yeast data is from 156 ChIP-chip experiments each measuring the binding of a single TF [14]. The mouse data is from 13 ChIP-seq experiments measuring binding of a TF [11]. The yeast TF data has been used extensively as a test case for evaluating motif discovery algorithms, so using it allows us to easily compare MEME with PSPs to a large number of other algorithms. Since ChIP-seq data is inherently of a higher quality than ChIP-chip data, the mouse TF data allows us to measure the benefit of using PSPs on a slightly easier motif discovery task. The mouse data covers 13 TFs–Nanog, Oct4, Sox2, Smad1, E2f1, Tcfcp2l1, Ctcf, Zfx, Stat3, Klf4, Esrrb, c-Myc and n-Myc.

We measure accuracy of MEME both with and without the use of PSPs. The PSP we use is the discriminative conservation prior (), which has previously been shown to be very effective for discovering TF binding site motifs in the yeast dataset [8]. The  prior is based on the degree to which the 8-mer starting at position *X*_*i*,*j *_is conserved in the input sequence set **X **and a set of orthologous sequences from other species, compared with a negative set of sequences and their orthologs. For comparison, we measure the accuracy of the PRIORITY motif discovery algorithm using the  PSP (PRIORITY-). We also compare with previously published results on the yeast dataset.

On the yeast data, we use the  PSP as reported by Gordân *et al *[8]. This prior is based on intergenic regions from *S. cerevisiae *that have a ChIP-chip fluorescence *p*-value ≤ 0.001 and the orthologous regions from the six related organisms *S. paradoxus*, *S. mikatae*, *S. kudriavzevii*, *S. bayanus*, *S. castelli*, and *S. kluyveri*. The negative sequences are all *S. cerevisiae *intergenic regions with a *p*-value ≥ 0.5 and their orthologs.

We create our own  PSP for each of the 13 mouse datasets. For each dataset, the positive sequences are 200 base pair (bp) regions centered on the ChIP-seq peaks reported by Chen *et al*. [11]. We use the mafFrags program to obtain orthologous sequences for sixteen additional species from the *multiz17way *alignment [15]. We obtain negative sequences and their orthologs for constructing the mouse  PSP by extracting 100 bp regions on either side of each positive sequence. We use the -PSP creation scripts provided by the Hartemink group [8] to create the mouse PSPs from the positive and negative sequence and ortholog sets. (More detail including the list of other species is in Additional file 1.)

To measure the accuracy of motif discovery on the yeast datasets, we utilize the same metric as previous researchers [8,14]. This metric compares the single motif reported by a motif discovery algorithm to a known motif for the TF by computing the scaled Euclidean distance between the PSPMs for the motifs. The distance is scaled so that the maximum distance is 1, and the minimum distance is 0. The scaled Euclidean distance between PSPMs *f *and *g *is defined as(6)

where *f*_*a, i *_and *g*_*a, i *_are the probabilities of base *a *at position *i *in the two motifs. We use the same known PSPMs as used by previous researchers [8], and the same criterion for successful motif discovery–scaled Euclidean distance *<*0.25. Since the reported motif may be of a different length or on the opposite DNA strand from the known motif, we actually compute the minimum value of *D *for all possible alignments of the reported motif (or its reverse complement) with the known motif, with the minimum overlap the length of the shorter motif.

Our evaluation of motifs discovered in the yeast ChIP-chip datasets utilizes a human-curated set of motifs that represents the consensus predictions of many motif discovery algorithms on those datasets. Such a "gold standard" set of motifs does not exist for the 13 mouse ChIP-seq datasets. Consequently, we take a different approach to measuring the accuracy of motifs discovered in those datasets.

With the mouse ChIP-seq datasets, our underlying measure of motif quality is the amount of correlation between a motif-based binding affinity score and a ChIP-based binding score. We believe that a high correlation between an *in vivo *measure of TF affinity and a motif-based *in silico *measure is indicative of an accurate TF binding motif. (We describe our two binding affinity scores and the correlation measure we use in the next paragraph.) For each ChIP-seq dataset, we measure this correlation in a cross-validation setting, discovering motifs on randomly chosen sets of positive and negative sequences, and computing the correlation measure on held-out sequences. To compare algorithms, we compare our correlation-based quality measure between motifs found on the same sample of sequences.

The details of our evaluation of motifs in the mouse ChIP-seq data are as follows. Our ChIP-based estimate of binding by the ChIP-ed TF at a genomic location is the "peak score" reported by Chen *et al. *[11], and is the normalized count of the number of sequence tags overlapping the peak's genomic location. This is our best direct evidence that the TF was bound in the neighborhood of the peak. Each positive sequence is assigned the peak score of the peak it contains. Our motif-based measure of binding by the ChIP-ed TF is for each positive sequence is its "Average Motif Affinity" (AMA) [16] score. The AMA score is justified as a measure of TF binding affinity on theoretical grounds [17], and it has been used for motif discovery [5] where it showed strong correlation with gene expression, and for motif enrichment analysis [18,19] where it showed strong correlation with TF binding. Because the AMA score estimates the average binding affinity of a region of DNA, it captures contributions from multiple sites in a given region. Our motif quality measure is the Spearman correlation coefficient (CC) between the ranks of the held-out positive sequences sorted by their AMA and peak scores, respectively. We use a rank-based statistic because it is less sensitive than a correlation between the original values to dissimilarities in the distributions being compared [20]. To compare pairs of algorithms, we use each algorithm to learn one motif in each of 50 random samples consisting of 100 positive sequences and 200 negative sequences. We then apply the sign test to the quality measures of pairs of motifs learned on the same input set to decide if one algorithm discovers significantly better motifs.

All yeast runs use a third-order Markov background model, for consistency with reported PRIORITY results. We let MEME search for a single motif of width from 7 to 12, with sites on either DNA strand. For all mouse runs, MEME and PRIORITY- use a fifth order background Markov model, computed from the negative set, and search for motifs of widths 8 to 20. To compute AMA, we use the AMA program, which is part of the MEME suite of programs [21], using the same background model as we use in motif discovery. In all cases, we use PRIORITY 2.0.0 with its default settings, except for changing to a fifth-order background model for the mouse runs. We test MEME with both the OOPS and ZOOPS models, with and without the  PSP, with sites on either strand.

## Results and Discussion

### Improved motif discovery using MEME with PSPs in yeast TF ChIP-chip datasets

Our evaluation of the effect of adding PSPs to MEME starts with measuring improvement in finding TF motifs in yeast ChIP-chip datasets. We run MEME using the OOPS and ZOOPS models with and without the  prior on each of 156 ChIP-chip datasets, and compare the single reported motif PSPM to the known PSPM for the TF pulled down in the ChIP-chip experiment. Success is defined as scaled Euclidean distance *<*0.25 between the reported PSPM and the known PSPM for the TF. Note that, to insure our results are directly comparable to the results reported by Gordân *et al. *[8], we use the script provided by them to compute the scaled Euclidean distance, which reports a distance of 1.0 (the maximum) if the found motif does not contain a region of width six with average information content at least 1 bit.)

The improvement in the number of motifs successfully discovered is quite dramatic. Using the  PSP with MEME more than doubles the number of yeast TF motifs successfully discovered (Table 2). The most successful approach is using MEME with the ZOOPS model with the  prior (ZOOPS-), which discovers the correct motif in 81 of the 156 datasets. Without the  prior, MEME with the ZOOPS model only discovers the correct motif in 39 of the datasets.

**Table 2:** Performance of motif discovery algorithms on yeast TF ChIP-chip datasets.

*Algorithm*	*Description*	*Successes*	*Proportion of Successes*
PhyloCon	local alignment of conserved regions	19	12%
PhyME	alignment-based; uses EM	21	13%
MEME_c	MEME run with non-conserved bases masked	49	31%
PhyloGibbs	similar to PhyME but uses Gibbs sampling	54	35%
Kellis *et al*.	alignment-based	56	36%
Converge	alignment-based; uses EM	66	42%
PRIORITY-	Gibbs sampler with conservation-based priors	69	44%
PRIORITY-	Gibbs sampler with discriminative conservation-based priors	76	49%

MEME: OOPS	MEME with OOPS model	36	23%
MEME: ZOOPS	MEME with ZOOPS model	39	25%
MEME: OOPS-	MEME with OOPS model and priors	73	47%
MEME: ZOOPS-	MEME with ZOOPS model and priors	81	52%
PRIORITY-	Gibbs sampler with discriminative conservation-based priors	69 (3)	44%

The table shows the number motifs (out of 156) successfully discovered by the named algorithms. The results in the top half of the table are taken from Gordân *et al*. [8]. Results in the bottom half are for new experiments performed by us. Each algorithm is allowed to report one motif, and success is declared if the scaled Euclidean distance to the known PSPM is <0.25. Proportions (out of 156) successes are rounded to the nearest integral percent.

The accuracy of motif discovery by several other algorithms using these same yeast TF ChIP-chip datasets and success metric has been reported previously [8,22], allowing us to compare our current results more broadly. As seen in Table 2, the success rate of ZOOPS- (81 motifs found) is substantially higher than a number of conservation-based EM or Gibbs sampler motif discovery algorithms (PhyloCon [22], PhyME [23], PhyloGibbs [1], Converge [22], PRIORITY-C [8]).

The ZOOPS- approach also performs at least as well on the yeast datasets as the Gibbs sampler-based algorithm PRIORITY, when PRIORITY is given the same  PSP as MEME. The developers of the PRIORITY algorithm (and of the PSP concept) reported a success rate of 76 out of 156 on the yeast datasets (result shown in Table 2 above the horizontal line). However, since Gibbs sampling algorithms are stochastic–their outputs vary from run to run–we wished to place error bars on PRIORITY-'s success rate. We therefore ran PRIORITY- ten times on each yeast dataset. The success rate varied from 65 to 74 correct motifs, with an average success rate of 69 (sd = 3), as shown in the last line of Table 2. The fact that we did not observe any PRIORITY- run with as high a success rate as previously reported [8] may be a result of the stochastic nature of the algorithm, or may be due to us using a more recent version of PRIORITY (Version 2.0.0).

### Improved motif discovery using MEME with PSPs in mouse TF ChIP-seq datasets

As an additional check on the value of using PSPs with MEME, we measure the improvement in TF motif discovery on 13 mouse TF ChIP-seq datasets. Our evaluation of mouse data is intended to demonstrate that the results generalize to a data set of different properties–a higher eukaryote, with sequence data derived from a different technology. We measure the correlation between the ChIP-seq peak score ranks of the sequences, and the AMA score ranks assigned using the discovered motif. To insure that this measurement is unbiased, we measure the correlation using held-out sequences, which are not used in discovering the motif. We compare *pairs *of motif discovery algorithms by sampling from all the sequence data (positive and negative), and applying a paired significance test (sign test) to the pairs of correlation scores.

On the mouse datasets, using the  prior improves the accuracy of the motifs discovered by MEME (see Table 3), although the improvement is slight compared to that seen in the yeast ChIP-chip datasets. The OOPS model with the  prior has significantly better accuracy than without the prior on 3 of the 13 datasets, and shows no significant difference in accuracy on the other 10, according to the sign test. Similarly, the  prior improves the ZOOPS on 4 of the 13 datasets, but degrades the performance on 3 datasets. MEME using the  prior finds better motifs for TFs c-Myc and n-Myc with both the OOPS and ZOOPS models. The motifs for three other TFs (Stat3, Zfx and E2f1) are improved using the  prior with one or the other of the two sequence models. These results indicate that using the  prior with MEME will likely improve the accuracy of TF motifs found in ChIP-seq data from higher eukaryotes.

**Table 3:** Performance of motif discovery algorithms on mouse TF ChIP-seq datasets.

	**OOPS-****vs. OOPS**				**ZOOPS-****vs. ZOOPS**				**OOPS-****vs. ZOOPS-**				**OOPS-****vs. PRIORITY-**			
TF	W	L	T	*p*-value	W	L	T	*p*-value	W	L	T	*p*-value	W	L	T	*p*-value
Nanog			×	3.4e-01		×		1.3e-03	×			7.7e-03	×			2.1e-09
Oct4			×	1.0e-01			×	3.4e-01	×			5.8e-07	×			4.5e-14
Sox2			×	1.6e-01		×		1.6e-02			×	4.4e-01	×			1.3e-03
Smad1			×	1.0e-01			×	1.6e-01			×	2.4e-01			×	1.6e-01
E2f1			×	2.4e-01	×			4.5e-05		×		4.5e-05			×	4.4e-01
Tcfcp2l1			×	1.0e-01		×		7.7e-03		×		1.6e-02	×			1.9e-11
Ctcf			×	4.4e-01			×	2.4e-01			×	4.4e-01	×			8.9e-16
Zfx			×	1.0e-01	×			1.3e-03			×	1.6e-01	×			2.2e-10
Stat3	×			3.3e-03			×	4.4e-01			×	6.0e-02			×	1.6e-01
Klf4			×	1.6e-01			×	6.0e-02			×	1.0e-01			×	1.6e-01
Esrrb			×	6.0e-02			×	6.0e-02	×			3.3e-03		×		4.5e-14
c-Myc	×			3.3e-02	×			3.3e-03			×	2.4e-01		×		4.5e-05
n-Myc	×			1.5e-04	×			4.5e-05	×			1.6e-02		×		1.6e-08

**Total**	3	0	10		4	3	6		4	2	7		6	3	4	

The table compares the relative accuracy of pairs of motif discovery algorithms. Relative accuracy is measured by the correlation on held out sets of sequences of the sequence ranks based on ChIP-seq peak scores versus the ranks based on the motif-based AMA score. A check in the "win" or "W" ("loss" or "L") column indicates that the motifs found by the first (second) algorithm had significantly better Spearman rank correlation, as judged by the sign test on the 50 random repeats (p-value < 0.05). A check in the "tie" or "T" column indicates that there was no significant difference. The "Total" line shows the totals using the sign test to judge significance. OOPS, ZOOPS, OOPS- and ZOOPS- refer to MEME with those models and with or without the  prior.

**Figure 1:**
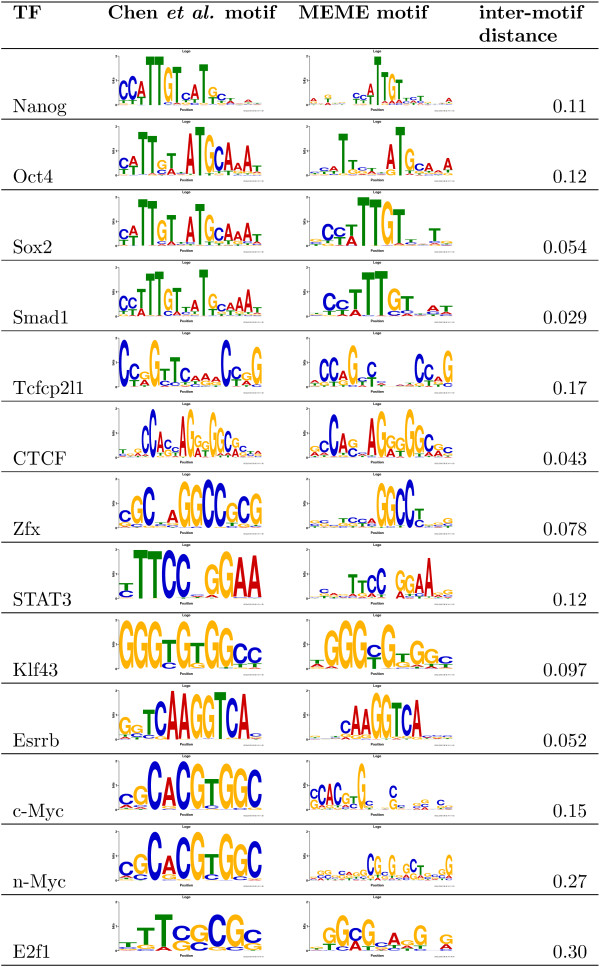
**Comparison of motifs found in mouse ChIP-seq datasets**. The figure shows the motifs reported by Chen *et al. *[11] and those found by MEME in sequences identified as bound to the given transcription factor in 13 ChIP-seq experiments. The MEME motifs were found using 100 randomly chosen bound sequences and the OOPS- prior. The inter-motif distance (scaled Euclidean distance) is computed as described in Additional file 1.

As a further evaluation of our method with ChIP-seq data, we also directly compare the motifs found by MEME using the OOPS- prior with those reported by Chen *et al. *[11]. In Figure 1, we show the Chen *et al. *motifs along side the motif found by MEME using a random sample of 100 ChIP-seq peak sequences that achieved the highest value of our unbiased correlation-based quality measure. For all 12 of the 13 Chen *et al. *ChIP-seq experiments where they reported a motif, MEME using the OOPS- prior discovers a strongly similar motif. Those authors reported no motif for the E2f1 experiment, but the motif found by MEME resembles the TRANSFAC [24] E2f1 motif. We also show the scaled Euclidean distance (Eqn. 6) between each Chen *et al. *motif and the MEME motif in Figure 1. (Note that we do not require the aligned motif regions to have average information content of at least 1 bit in the inter-motif distance computation in Figure 1. Without this change, the inter-motif distances for Oct4, n-Myc and E2f1 would be reported as "1.0".) All 13 motifs discovered by MEME have distances less than or equal to 0.30 to the corresponding Chen *et al. *or TRANSFAC motif, and 11 out of 13 have distances below 0.17. We emphasize however, that this result only indicates that MEME is finding motifs similar to those found by those authors, and we believe that our correlation-based quality measure is more appropriate with this data.

To answer the question of whether using the  prior with the OOPS or ZOOPS model is more appropriate with the mouse ChIP-seq data, Table 3 shows the relative accuracy of OOPS- versus ZOOPS-. According to the sign test, OOPS- finds significantly more accurate motifs in more datasets (4 vs. 2) than ZOOPS-. Although the sample size is small (13 datasets), it seems reasonable to conclude that the OOPS- approach will produce better motifs on average with ChIP-seq data.

A direct comparison of the accuracy of motifs found in the mouse datasets by OOPS- and PRIORITY- indicates that MEME with using the  prior and the OOPS model has a slight edge. According to the sign test, OOPS- produces significantly better motifs for 6 of the 13 mouse ChIP-seq datasets, compared with PRIORITY-. On 3 of the datasets, PRIORITY- produces more accurate motifs. This result is in agreement with our results using the yeast ChIP-chip datasets, where MEME using the  prior and the ZOOPS model was (slightly) more successful than PRIORITY-. As we expect, the ZOOPS model works better for ChIP-chip data, while the OOPS model works better for ChIP-seq for the examples we present here.

## Conclusions

Position specific priors are an elegant and flexible way to utilize prior information from heterogeneous sources to improve the discovery of sequence motifs. In addition to allowing information from multiple sources to be incorporated into a Bayesian motif discovery framework, positional priors can even incorporate information from negative examples (so-called "discriminative" priors). Furthermore, using PSPs does not increase the running time of the underlying motif discovery algorithm. This flexibility has the potential to extend the range of applications and sensitivity of motif discovery algorithms that can utilize PSPs. Although we only study DNA datasets, our modifications to MEME are not DNA-specific. MEME is freely available for academic use and downloading at http://meme.nbcr.net.

PSPs had previously been shown to be of benefit when used with a Gibbs sampling motif discovery algorithm. We have shown here that they can also be of great benefit to MEME, which is based on EM and a heuristic search for starting points. We focused on using a prior that combines evolutionary information gleaned from orthologous sequences with positively and negatively labeled sequences in a discriminative prior (the "discriminative conservation",  prior). Using this PSP on well-studied sequence datasets from 156 yeast TF ChIP-chip experiments improves the performance of MEME dramatically–more than doubling the number of datasets where MEME identifies the correct TF binding motif as its first prediction. Furthermore, using the  prior allows MEME to achieve prediction accuracies that are superior to a large number of motif discovery algorithms, without increasing its running time.

We also confirm the benefit of PSPs to MEME when applied to TF motif discovery in ChIP-seq data from a higher eukaryotic species (mouse). To increase the independence of this second test, we used a novel way to measure the accuracy of the discovered motifs that obviates the need to rely on a set of known motifs (a "gold standard"). Although the observed benefits were somewhat small, they were substantial enough to indicate that constructing a  prior and utilizing it with MEME is worthwhile even for higher eukaryotic ChIP-seq derived data.

In follow-up work, we plan to investigate PSPs designed specifically for ChIP-seq data. One approach might be to create a PSP that encodes the increased prior probability of the primary motif being located near the center of the ChIP-seq peak. We also plan to investigate PSPs designed for motif discovery in protein sequences. For protein motifs, PSPs based on spaced triples rather than the *k*-mers used here for DNA PSPs might be more appropriate, given the larger protein alphabet. We also intend to implement PSPs for use with MEME's ANR model, which allows multiple repeats of a motif within a single sequence. We don't foresee any major difficulties in incorporating PSPs into the ANR model but have focused on the OOPS and ZOOPS models in this work in order to facilitate direct comparison with previous work by others on PSPs.

## Authors' contributions

TLB designed the experiments, adapted the MEME algorithm to use PSPs and wrote the final draft. MB worked on the mouse evaluation method. TW assisted with processing the ChIP-seq data. PM did most of the programming, performed the experiments and wrote the initial draft.

## Supplementary Material

Additional file 1 Additional details on algorithm implementation and methods of evaluation. Click here for file
