# Supplementary material for: The value of position-specific priors in motif discovery using MEME
Source: BMC Bioinformatics. 2010 Apr 9;11:179. doi: 10.1186/1471-2105-11-179 (PMC2868008; doi:10.1186/1471-2105-11-179)
Supplement: Additional file 1 — Additional details on algorithm implementation and methods of evaluation. [file 1471-2105-11-179-S1.PDF]

Supplementary Material
The value of position-specific priors in motif discovery using MEME

Timothy L. Bailey, Mikael Bodén, Tom Whitington and Philip Machanick

February 10, 2010

1 Introduction

This supplementary material includes further detail of methods to support repeatability, and an expansion of the theoretical basis of the approach to adding position-specific priors (PSPs) to MEME. We provide additional detail of the data sets, including our approach to creating priors (for the mouse data; we use existing priors for the yeast data).

The remainder of this document is organized as follows. The first section describes the availability of the software and data described. The Section 3 provides more detail of the theoretical basis of our approach to adding PSPs to MEME. In section 5 we detail the data sets use and expand on the methods we use to obtain priors for mouse data and our evaluation strategy in Section 6.

2 Availability

The features described here are available in MEME from version 4.2.0 onwards, which can be obtained following the "Downloads" link at `http://meme.nbcr.net`. The downloadable software includes MEME, as well as a script (`harteminl2psp`) for converting PSPs from the format generated by software the Hartemink group [Gordân *et al.*, 2008] to the format used by MEME.

The ChIP-chip data for yeast transcription factors from the Harbison group [Harbison *et al.*, 2004], position-specific priors and the set of known TF binding motifs is contained in the compressed tar-file `yeast_suppl_data.tgz`.

3 Theory

In this section we provide some extra details of the theoretical basis for adding PSPs to MEME. We also recapitulate some details from previously published descriptions of $\mathcal{DC}$ priors, as an aid to understanding our approach to generating new priors for our mouse examples [Gordân *et al.*, 2008].

We use definitions of Alexander Hartemink's priors [Gordân *et al.*, 2008] as a starting point. We reproduce their definitions here for convenience. The first of these is *conservation score*:

$$\mathcal{S}_{\mathcal{C}}(\boldsymbol{X}_i, j) \quad = \quad \frac{1}{k}\sum_{s=1}^{k} I[\boldsymbol{X}_{i,j}^{W} \in \boldsymbol{X}_i^{(s)}] \qquad (1)$$

where I is an indicator function (1 if the given element is in the set, 0 otherwise). The reverse complement of a W-mer is counted as a match too, as illustrated in Fig. 1. In this example, if the two matches to the sequence at $\boldsymbol{X}_{1,2}$ of length W=5 shown are the only ones found in $\boldsymbol{X}_{i,j}^{(s)}$, then $\mathcal{S}_{\mathcal{C}}(\boldsymbol{X}_i, j)$ in this instance is $\frac{2}{k}$. Orthologous sequences $\boldsymbol{X}_i^{(s)}$,

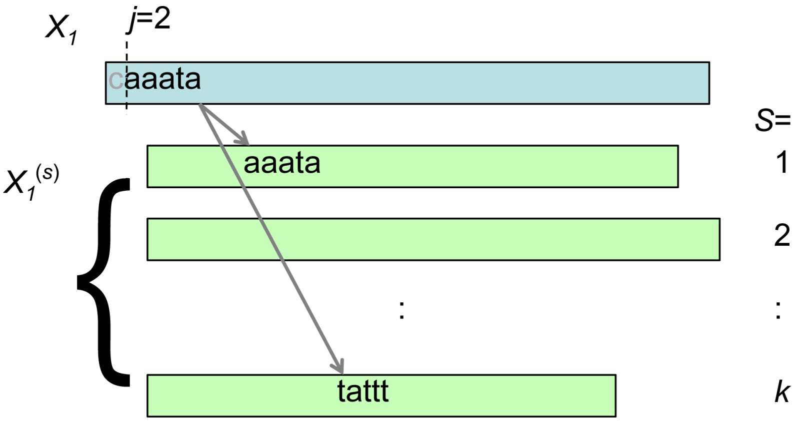

Figure 1: Conservation score example.

therefore, are used to derive this conservation score. $\mathcal{S}_\mathcal{C}(\boldsymbol{X}_i, j)$ in effect is a weighed (by $\frac{1}{k}$) count of how often a particular subsequence or W-mer at position j in sequence $\boldsymbol{X}_i$ occurs in any of the k orthologs. This score will be at most 1 (if all k orthologs contain at least one instance of $\boldsymbol{X}_{i,j}^W$) and would be 0 if the W-mer was absent from all orthologs.

A *discriminative score* uses the same formula as the conservation score, but uses a *negative* sequence set $\boldsymbol{Y}$ based on sequences that are considered unlikely to contain the motif. From the conservation and discriminative scores, a *discriminative conservation score* is computed:

$$\mathcal{S}_{\mathcal{DC}}(\boldsymbol{X}_i, j) = \frac{\sum_{(q,r):\boldsymbol{X}_{qr}^W = \boldsymbol{X}_{i,j}^W} \mathcal{S}_\mathcal{C}(\boldsymbol{X}_{q,r})}{\sum_{(q,r):\boldsymbol{X}_{qr}^W = \boldsymbol{X}_{i,j}^W} \mathcal{S}_\mathcal{C}(\boldsymbol{X}_{q,r}) + \sum_{(q,r):\boldsymbol{Y}_{qr}^W = \boldsymbol{X}_{i,j}^W} \mathcal{S}_\mathcal{C}(\boldsymbol{Y}_{q,r})} \qquad (2)$$

The idea of the $\mathcal{S}_{\mathcal{DC}}$ score is to distinguish W-mers that are conserved only in the bound set from those more generally found throughout the genome. The logic is that since a CHiP-chip (or similar) experiment found that the TF bound in the set $\boldsymbol{X}$ but not in $\boldsymbol{Y}$, any promising-looking motif found in $\boldsymbol{X}$ should be given less weight if it also occurs in the negative set. The Hartemink approach to defining the positive $\boldsymbol{X}$ set is to choose sequences found in the intergenic region of yeast (*S. cerevisiae*) which bind to a particular TF with p-value ≤ 0.001, while $\boldsymbol{Y}$ is defined to be intergenic sequences bound with a p-value ≥ 0.05. We return to the matter of defining the positive and negative set when considering how to compute our own priors with a different data set (§5.2).

4 Implementation in MEME

Position-specific priors can be provided to MEME via the newly-added command line switch "`-psp <psp_file>`". The PSP file specifies a position-specific prior for a corresponding FASTA sequence dataset. The entries in the PSP file are similar to FASTA format, except the sequence letters are replaced by floating point numbers.

A PSP file entry begins with a header line containing "`>seq_name width`", where "`seq_name`" must match a sequence name in the sequence file associated with the PSP file, and "width" is the motif width associated with the PSP. The width should be the same for all PSP file entries. Following the header line, and starting on a new line, the actual PSP values should be listed, separated by white space. If the associated sequence has L letters, there should be L PSP values. The ith entry represents the prior probability of a motif site at position i in the associated sequence. A PSP is assumed to be symmetrical in the case of searching both strands of DNA (using the "`-revcomp`" option). The last w-1 positions in the PSP file should be zeros, reflecting the fact that MEME will only find sites for a motif that fits entirely in the sequence data. A short example follows:

Transcription Factor	number of sequences
Ctcf	38238
E2f1	19856
Esrrb	20874
Klf4	10509
Nanog	9290
Oct4	3560
Smad1	1038
Sox2	4046
Stat3	2380
Tcfcp2l1	25475
Zfx	10093
c-Myc	3355
n-Myc	7031

Table 1: **Number of sequences for mouse data**. The number of sequences per TF varies from just over 1,000 (Smad1) to over 38,000 (Ctcf).

```
>A2ACQ1 3
0.15 0.16 0.13 0.22 0.19 0 0
```

Depending on the software used to generate the PSP file, there may be additional information on the header line. In this example, the sequence that this PSP corresponds to is named "`A2ACQ1`", the PSP is computed for a word of length 3 (shorter than the length of 8 used in the paper). The PSP data contains 7 numbers and therefore is for a sequence of length 7 (again shorter than any real example we work with). Since w_0 = 3, there must be two zeros at the end of the PSP, representing sites that cannot be starting points for a motif of length 3. The PSP in this example adds up to 0.85, indicating that this is a ZOOPS PSP with a probability of not finding a site of 0.15 (i.e., $P_{1,0} = 0.15$ in our notation). In a real example, we would usually have PSPs for multiple sequences in the same file, each starting with a new header line.

5 Data Sets

5.1 Yeast Sequences

The yeast (*S. cerevisiae*) data we use is sequences for 156 transcription factors (TFs) obtained from ChIP-chip experiments [Harbison *et al.*, 2004], used in the original $\mathcal{DC}$ priors paper [Gordân *et al.*, 2008]. We reuse the $\mathcal{DC}$ priors computed by Gôrdan *et al.* and also borrowed their evaluation methodology (scaled Euclidean distance relative to known motifs, as reported in the main body of the paper).

5.2 Creating Mouse Sequences and Priors

Our mouse data is based on ChIP-seq experiments [Chen *et al.*, 2008], with sequences covering for TFs (Tab. 1). We widen each sequence to 200 base pairs centred on the original locus (the original sequences were all less than 200 base pairs long). Since ChIP-seq is a newer technology than ChIP-chip, the sequences have lower variance in specificity to each TF, so identifying a negative set out of the sequences is not an option. Instead, our approach is to take sequences adjacent to each positive sequence (the set $\boldsymbol{X}$), as described in the main body of the paper. The logic is that we want the negative set ($\boldsymbol{Y}$) to be similar in general properties to the positive set, except that it should not contain binding sites. Our background model for mouse data is also based on this negative set, $\boldsymbol{Y}$.

Our starting point is data in the following format, as used by Chen *et al.*:

```
<chr#>:<start>-<end> <count lib> <count control>  <peak value>
```

species	date	version
mouse	Feb. 2006	mm8
rat	Nov 2004	rn4
rabbit	May 2005	oryCun1
human	Feb 2006	hg18
chimp	Jan 2006	panTro2
macaque	Jan 2006	rheMac2
dog	May 2005	canFam2
cow	Mar 2005	bosTau2
armadillo	May 2005	dasNov1
elephant	May 2005	loxAfr1
tenrec	Jul 2005	echTel1
opossum	Jan 2006	monDom4
chicken	Feb 2004	galGal2
frog	Aug 2005	xenTro2
zebrafish	Mar 2006	danRer4
Tetraodon	Feb 2004	tetNig1
Fugu	Aug 2002	fr1

Table 2: **Orthologous species for mouse priors**. Based on the `multiz17way` track.

Where `<count lib>` is the count of occurrences in the ChIP library and `<count control>` is the count in the control library. The `<peak value>` represents fold-change versus the negative control library normalized for sequencing depth. We use the start and end positions to create sequences, and use the peak value in our motif evaluation (§6.3). For example, one line of the file representing Nanog sequences looks like this:

```
chr1:3053032-3053034    53      1       11.469303
```

and the corresponding FASTA sequence, widened to 200 base pairs (noting that the BED file format numbers the final position as one past the end of the sequence) is:

```
>mm8_Nanog_chr1_0003052933_0003053133|mm8 11.469303
TTGGTGCCTGTTGCCAGCCTGTAGGTGATTTGCTGGATACTGTCTCCTGAATATCTCAAGGGCCTCTCAGCAGGTAG
CAGTGTCTTGGAGAACAGCAACAGTTGACAGAACAATAGAGCCATTTAAATTGGAAGGTTCCACCCCAGGTGGTCTC
ATTCTCAGTGGCACCAAGGACTAAGCCAGCCTACTCAAGGCTGGGG
```

To obtain sequence data and find orthologous sequences for purposes of computing priors, we use the `mafFrags` program[1]. As an additional step, we first convert the Chen files to BED format, as required by `mafFrags`. At the same time we widen the sequences to 200 base pairs. Using the same inputs, we create a BED file representing sequences a fixed offset from the given loci; we use this latter feature to create the negative set, $\boldsymbol{Y}$. We use the `mm8` mouse database (February 2006 mouse genome data) and the `multiz17way` track containing 17-way alignments with mouse (the species are detailed in Table 2). We enclose these steps in a script that takes each line of the BED file, one at a time, and uses `mafFrags` to find the main species and orthologous sequences. This outer script converts the output of `mafFrags` to FASTA format. In summary:

1. widen loci and convert the Chen data format to BED format, keeping the peak value in the name line of each sequence
   (a) do this for 200 base pairs centred on the original locus for each sequence forming the positive set $\boldsymbol{X}$
   (b) do this twice for 100 base pairs on either side of the 200 base pairs for each positive sequence, forming the negaitive set $\boldsymbol{Y}$ (note that $\boldsymbol{Y}$ has twice as many sequences as $\boldsymbol{X}$ but each negative sequence is half the length of a positive sequence)

[1] `http://genomewiki.ucsc.edu/index.php/Kent_source_utilities`

2. run each BED file through `mafFrags` to create sequence data for the main species plus orthologs
3. combine the above two steps, taking the BED data one line at a time, to create FASTA files out of the output from `mafFrags`

Here is a sample command line for `mafFrags` (the final name on the line contains the output):

```
mafFrags mm8 multiz17way fillename.bed filename.maf
```

Given the positive and negative sets and their orthologs, we run scripts supplied by Alexander Hartemink to create $\mathcal{DC}$ priors. We also use the negative set to compute a 5-th order background Markov model, and use the positive set to find motifs and to evaluate the quality of the found motifs, as described below (§6.3).

6 Methods

In this section we describe how we use the data sets and describe the scripts we use to obtain results and perform evaluations of those results. We also provide some additional details of the AMA calculation.

6.1 Running on Yeast ChIP-chip Data

To run on yeast data, we use a 3rd-order Markov background model we derived from that supplied with PRIORITY (obtained from its authors [Gordân *et al.*, 2008]), which we converted to MEME's data format. We resuse the background model used for earlier yeast results to limit the number of variables in our comparative study. We run each instance of MEME with the following command-line parameters, plus variations as described below:

```
TFname.fasta -minsites 20 -dna -revcomp -minw 7 -maxw 12 -bfile BKG
```

where `TFname` is the TF for which the sequences were generated and `BKG` is the background file. For OOPS runs, we add the following to the command line:

```
-mod oops
```

For PSP runs, we add:

```
-psp PSPfile
```

where `PSPfile` is the file containing PSPs for the TF represented by the FASTA file.

We run PRIORITY-$\mathcal{DC}$ using a command-line interface since we use scripts to run multiple instances:

```
java -jar priority.jar -nogui
```

where the PRIORITY application `priority.jar` may need a path appended to its name. PRIORITY picks up its options from a configuration directory. We run with defaults except to change the names of the input and output files.

6.2 Running on Mouse ChIP-seq Data

For mouse data, we use a 5th-order Markov background model derived from the negative sequence set, $\boldsymbol{Y}$. We run each instance of MEME with the following command-line parameters, plus variations as described below:

```
TFname.fasta -minsites 20 -dna -minw 8 -maxw 20 -revcomp -bfile BKG
```

where `TFname` is the TF for which the sequences were generated and `BKG` is the background file. For OOPS runs, we add the following to the command line:

```
-mod oops
```

For PSP runs, we add:

```
-psp PSPfile
```

where `PSPfile` is the file containing PSPs for the TF represented by the FASTA file.

We run PRIORITY in its command line version, with its default settings, except for input and output file names, with one exception: we set the background model to order 5, to match the background model used in MEME.

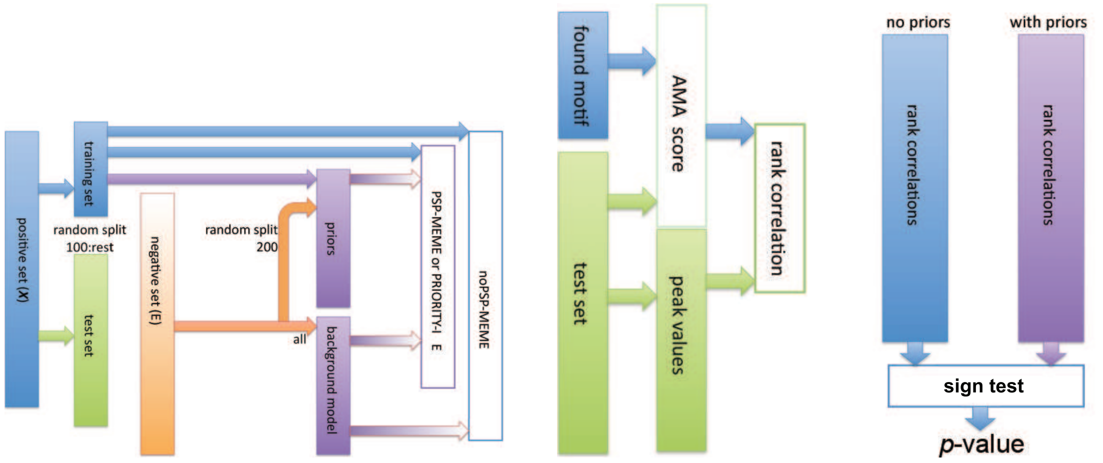

(a) main data flows (b) correlations (c) sign test

Figure 2: **Cross validation**. We do 50 independent random splits of the data for each transcription factor, and correlate the AMA and peak scores arising out of comparing a found motif with held-out test sequences. Once we make a random split, we do not use the held out test set at all until we use it to test a found motif using the correlation measure. This cross-validation approach ensures that we are not over-fitting or biasing our scores, because we use disjoint training and test data. We illustrate the sign test here as between no priors and with priors; we generalise this step to comparing any pair of algorithms.

6.3 Evaluation Methodology

Our approach to evaluation of yeast and mouse data differs. For the yeast TFs, existing motifs are available to use as a "gold standard" [Harbison *et al.*, 2004]. We therefore are able to compare our motifs with those literature motifs, as described in the paper. For evaluating outputs from mouse runs, we do not have a "gold standard" and instead compare outputs of competing algorithms using a form of cross-validation:

1. We split the positive $\boldsymbol{X}$ sequences into two sets, the *training* set consisting of 100 randomly selected sequences for each TF, and the *test* set, consisting of the remainder of the sequences.

2. We similarly split the negative set $\boldsymbol{Y}$ into a training set of 200 sequences (twice as many as for the positive set because the sequences are each half the length); in this case there is no test set, since the negative sequences play no role once we find a motif.

3. We use the training set to derive $\mathcal{DC}$ priors, as well as to run both MEME and PRIORITY-$\mathcal{DC}$.

4. We use the test set to provide an evaluation measure as follows.

   (a) for each motif we have found using PRIORITY-$\mathcal{DC}$ and the various variations on MEME (with and without PSP for each of OOPS and ZOOPS), we run AMA on the found motif, testing it against the test set. This gives us a single number for each sequence in the test set, for a given motif.

   (b) we also extract the peak value from the original mouse data set for each element of the test set.

   (c) we now have two numbers for each member of the test set: an AMA score (specific to the motif we found) and a peak score (derived from the ChIP-seq data and independent of any algorithm we have run): we correlate the *ranks* of these two sets of numbers.

5. The resulting rank correlation coefficient (CC) becomes a basis for comparing different algorithms run on the same data set.

6. To provide a statistically significant measure, we repeat the above steps for a total of 50 random splits of the data, and use the sign test to determine whether there is a signficant difference between any pair of algorithms.

The AMA score for a sequence given motif θ_M is calculated as

$$AMA(s) = \frac{1}{m} \sum_{i=1}^{m} \prod_{j=0}^{w-1} \frac{P(s_{i+j}|\theta_M)}{P(s_{i+j}|\theta_B)}, \quad (3)$$

where $P(s_{i+j}|\theta_M)$ is the probability of observing the symbol at position $i + j$ in the sequence given motif model θ_M, and $P(s_{i+j}|\theta_B)$ is the probability of the symbol under the background model, θ_B. To compute this, we use the AMA program, which is part of the MEME suite of programs [Bailey *et al.*, 2009].

Fig. 2 illustrates the major data flows. Fig. 2(a) shows how the original sequence data was split into *test* and *training* sets. The test set plays no role in finding motifs. The rank correlations (Fig. 2(b)) compare a value derived from the found motif compared against the test set (the AMA score), and a value totally independent of the found motif, the peak value. We use the sign test (null hypothesis: PSPs make no difference) to evaluate the statistical significance of the number of times one of a pair of competing algorithms has a higher CC, computed for 50 different random splits.

6.4 Speed

Adding PSPs has no measurable effect on run time of MEME; the major effect on speed is the time taken to compute PSPs. Since PSPs are an additional input to MEME and the same data can potentially be used as for other programs, run time to compute PSPs is not a focus of this paper.

We do not make specific speed claims versus PRIORITY, since the approach we use for the mouse data of running multiple small examples favours an algorithm with higher time complexity based on data set size. MEME has run time $O(N^2)$ where N is the total data set size. PRIORITY on the other hand is claimed to have run times that vary insignificantly with the number of sequences [Gordân *et al.*, 2008]. On mouse examples, PRIORITY ran about 2.5 times slower than MEME but users of PRIORITY may choose a different approach than ours, where its differing time complexity would confer an advantage.

References

[Bailey *et al.*, 2009] Timothy L Bailey, Mikael Boden, Fabian A Buske, Martin Frith, Charles E Grant, Luca Clementi, Jingyuan Ren, Wilfred W Li, and William S Noble. MEME Suite: tools for motif discovery and searching. *Nucleic Acids Res*, 37(Web Server issue):W202–W208, Jul 2009.

[Chen *et al.*, 2008] Xi Chen, Han Xu, Ping Yuan, Fang Fang, Mikael Huss, Vinsensius B. Vega, Eleanor Wong, Yuriy L. Orlov, Weiwei Zhang, Jianming Jiang, Yuin-Han Loh, Hock Chuan Yeo, Zhen Xuan Yeo, Vipin Narang, Kunde Ramamoorthy Govindarajan, Bernard Leong, Atif Shahab, Yijun Ruan, Guillaume Bourque, Wing-Kin Sung, Neil D. Clarke, Chia-Lin Wei, and Huck-Hui Ng. Integration of external signaling pathways with the core transcriptional network in embryonic stem cells. *Cell*, 133(6):1106–1117, Jun 13 2008.

[Gordân *et al.*, 2008] Raluca Gordân, Leelavati Narlikar, and Alexander J. Hartemink. A Fast, Alignment-Free, Conservation-Based Method for Transcription Factor Binding Site Discovery. In Martin Vingron and Limsoon Wong, editors, *12th Annual International Conference on Computational Biology, RECOMB 2008*, pages 98–111. Springer-Verlag, March 2008.

[Harbison *et al.*, 2004] Christopher T Harbison, D. Benjamin Gordon, Tong Ihn Lee, Nicola J Rinaldi, Kenzie D Macisaac, Timothy W Danford, Nancy M Hannett, Jean-Bosco Tagne, David B Reynolds, Jane Yoo, Ezra G Jennings, Julia Zeitlinger, Dmitry K Pokholok, Manolis Kellis, P. Alex Rolfe, Ken T Takusagawa, Eric S Lander, David K Gifford, Ernest Fraenkel, and Richard A Young. Transcriptional regulatory code of a eukaryotic genome. *Nature*, 431(7004):99–104, Sep 2004.
